# Dr. Benjamin Lee

**Published:** 1891-03

**Authors:** 


Dr. Benjamin Lee, Secretary of the State Board of Health of Pennsylvania, has accepted the position of Secretary of the Section on State Medicine of the American Medical Association.

As the meeting takes place in Washington, May 5th, it is important that all papers intended for this section should be in his hands by the fifth of April. All members of the association desiring to be enrolled in the Section are requested to forward him their names at 1532 Pine St., Philadelphia.

